# Fifteen to twenty-five year functional outcomes of twenty-two patients treated with posterior Cotrel-Dubousset type instrumentation: a limited but detailed review of outcomes

**DOI:** 10.1186/s13013-016-0079-6

**Published:** 2016-07-28

**Authors:** William F. Lavelle, Andy A. Beltran, Allen L. Carl, Richard L. Uhl, Khalid Hesham, Stephen A. Albanese

**Affiliations:** 1 Department of Orthopedic Surgery, SUNY Upstate Medical University, 750 East Adams Street, Syracuse, NY USA; 2 Albany Medical College, 43 New Scotland Avenue, Albany, NY USA; 3 Albany Medical Center, Division of Orthopaedic Surgery, 1367 Washington Ave., Suite 200, Albany, NY USA; 4 Albany Medical Center, Albany, NY USA; 5 Present address: 1330 1st Ave, Apt 1203, New York, NY USA; 6 6620 Fly Road, Suite 200, East Syracuse, NY 13057 USA

**Keywords:** Adolescent idiopathic scoliosis, Long-term functional outcomes, Cotrel-Dubousset, Back pain, Visual analog scale, Oswestry Disability Index (ODI), Short-Form 36 (SF-36), Scoliosis Research Society 22 (SRS-22)

## Abstract

**Background:**

Long-term outcomes of patients undergoing extensive fusions for adolescent idiopathic scoliosis (AIS) have conflicting results. Moskowitz found uninstrumented scoliosis fusion patients had similar back pain as a normal age matched population. Recently, long-term outcomes of patients with Harrington rod instrumentation were reviewed and found similar functional outcome scores to non-scoliosis patients, with trending toward worse outcomes when fusions extended to L4. Our study examined long-term functional outcomes of patients treated with Cotrel-Dubousset (CD) instrumentation and determined whether distal level of instrumented fusion (L4 and L5) correlate with increased back pain or lower functional level.

**Methods:**

Retrospective review of AIS surgeries from 1986 to 1996 was undertaken. Patient demographics and surgical data were collected via case-note audit. Patients were contacted and asked to complete a series of functional outcome questionnaires including visual analog scales (VAS) for pain, Short-Form 36 (SF-36), Scoliosis Research Society 22 (SRS-22) and Oswestry Disability Index (ODI) for function. ANOVA technique categorically compared outcome scores to most distal levels of fusion. Linear regression compared patient reported outcomes to time elapsed since surgery. Statistical significance was *p* < 0.05.

**Results:**

One hundred twelve patients were identified, 50 patients were contacted, and 22 agreed to participation and completed a full assessment. Follow-up time since surgery ranged from 15 to 26 years and age ranged from 30 to 43 years. Six patients reported daily VAS back pain of ≥5; with a mean of 2.5. Back pain was not associated with level of distal fusion (*p* = 0.92). ODI was 15.36, with six patients' ODI >20. No relationship was shown between ODI and distal level of fusion (*p* = 0.72). SF-36 and SRS 22 values were also not related to distal level of instrumentation. Patient reported VAS back pain scores (r^2^ = 0.18, *p* = 0.05), ODI (r^2^ = 0.09, *p* = 0.17), and SF-36 and SRS-22 were not worse in patients with longer follow-up over time. Back pain and certain functional score subcategories of the SF-36 and SRS-22 trended toward improved results over time.

**Conclusions:**

Most patients who underwent multi-segment spinal fixation appeared to do well long-term, with minimal back pain. Lowest instrumented segment did not appear to be associated with increased back pain after 15 to 25 years follow-up.

## Background

The origin of surgical treatment for adolescent idiopathic scoliosis (AIS) dates back to 1911 when Hibbs [1] first described his spinal fusion procedure on three patients with Pott’s disease. Outcomes in the early 1900s compared solid fusions to pseudarthroses. The Harrington rod [2] became the most widely used surgical treatment from the 1960’s through the early 1990’s, being used in over 85 % of scoliosis patients [3]. In 1984, Cotrel and Dubousset [4] introduced a segmental technique which provided the foundation for the procedures and instrumentation widely used today. With each new procedure and instrumentation came an improved ability to provide three dimensional spine corrections. Many outcome studies have traditionally looked at the success of these treatments by focusing on the degree of spine correction its ability to halt curve progression, and complication rates. However, it has been noted that the degree of spine correction has not directly correlated with patient satisfaction [5].

Recently, investigators have critically looked at outcome scores to assess the quality of patient outcomes for long posterior spinal fusions for AIS. In the 1970s, the results of fusion in Risser’s original cohort, who had been followed for over 34 years, were reviewed [6]. Risser’s cohort of eight patients treated by cast correction and fusion showed maintenance of fusion and no symptoms of back pain. This was followed by Moskowitz et al. [7] who looked at outcomes of patients who had been fused over the previous 25 years and these also demonstrated similar results with a larger patient population. They specifically analyzed quality of life, and concluded that patients had no more pain than the general population. In 1983, Cochran [8] reported on the long-term results of patients who had long scoliotic fusions into the lumbar spine with Harrington rod instrumentation with a minimum of 5 years follow-up. Cochran found greater than 75 % of patients fused to L4 and L5 had significant pain, while only 25 % of those patients fused to L1 had pain. Other authors have examined intermediate radiographic results such as the durability of deformity correction and the extent of degenerative change noted radiographically [9].

In contrast, Bartie et al. [10] reviewed the long-term outcomes of patients who had Harrington rod instrumentation for AIS with a minimum of ten year follow-up. They specifically looked at patients who were fused to various levels in their lumbar spine and found back pain was higher among the surgical population when compared to a control group of similar sex, age, height, weight, and scoliosis type. Though not statistically significant, there were no differences seen between patients fused to the upper (L2-3) versus lower lumbar spine (L4-5). When looking at “intense” back pain, this study showed an increase of pain with distal lumbar fusion and instrumentation. The reported data on long-term analysis of posterior fusion and instrumentation is scarce and at times contradictory.

Although the Cotrel-Dubousset (CD) instrumentation has been utilized for almost 30 years, there are only a few mid-term studies and no long-term studies on patient functional outcomes after CD instrumentation. The purpose of our study was to examine the long-term functional outcomes of patients treated with CD instrumentation. We believe it is important to critically evaluate patient functional outcome scores and determine how AIS treated with modern instrumented fusion techniques affects the quality of life. Our specific aim was to determine whether the distal level of instrumented fusion (L4 and L5) correlated with increased back pain or lower functional level.

## Methods

This study was performed with the approval of our institutional review boards (IRB) at Albany Medical Center and the State University of New York Upstate Medical University and in accordance with the boards’ ethics regulations. After IRB approval, the AIS surgeries that were completed between 1986 and 1999 by the senior author (SA) were examined. The author had treated the majority of the AIS patients in the Upstate, New York (Syracuse) area. These patients were seen, evaluated, and treated according to the standard care of practice. All patients underwent a complete history and physical examination along with standing x-rays; and later, went on to have instrumented spinal fusions completed using three dimensional instrumentations according to the CD technique with posterior only spinal fusions.

In a retrospective manner, all patient demographic data were collected to include: age, sex, race, skeletal maturity determined by Risser Classification, type, and magnitude of curve determined by the Cobb Measurement. This data was collected from the medical chart reporting the initial history and physical examinations as well as from the initial x-rays taken at the time the patients presented to the offices. The levels selected for spinal fusion were reviewed; and specifically, the most distal level selected for this fusion procedure was noted. Additionally, we recorded whether further extension of the initial instrumented fusion was performed at a later date. From this data collection, a review of records was performed prior to contacting patients for interview. This was to ensure the subjects were at least 18 years of age at this time for purposes of data collection. We also ensured that the contacted patients had undergone surgery for their scoliosis while under the age of 18. There were no other inclusion/exclusion criteria.

If the subjects agreed to be contacted and provided phone numbers, a study investigator interviewed the patients and asked explicitly if they had any current back pain, had undergone any subsequent spinal surgery, and, if they would be willing to submit written questionnaires. The questionnaires sent contained: Oswestry Disability Index (ODI); Short-Form 36 (SF-36); Scoliosis Research Society 22 (SRS-22), and a horizontal VAS for back [11] Patients were asked to mark their average back pain from zero to ten.

Outcomes reported by these patients were categorized by extent of distal fusion (coded as 1 = L4, 2 = L3, 3 = L2 or above) and compared. Statistical analysis was completed using a Chi-square technique for categorical variables, and ANOVA technique for categorically comparing the patient reported outcomes to the most distal level of fusion. Linear regression compared patient reported outcomes to the time elapsed since surgery. Statistical significance was accepted at *p* < 0.05.

## Results

One hundred twelve patients were identified who met all inclusion criteria to include posterior only CD constructs and identifiable contact information. Fifty of the 112 patients were able to be contacted via a telephone interview, and 62 patients could not be contacted via telephone as they did not have a working phone number on file or refused the phone call. Twenty-two of the 50 agreed to complete the full assessment of outcome scores. The follow-up time since surgery ranged from 15 to 26 years (mean 20 years). Follow-up patient age ranged from 30 to 43 years of age (mean 35 years).

**Fig. 1:**
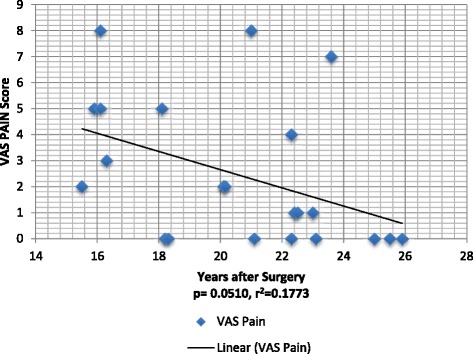
VAS Back Pain versus time since surgery

On the horizontal VAS survey, six of the 22 patients reported daily back pain of 5 or greater (Fig. 1). The mean of the reported VAS back pain was 2.5 in the study population (Table 1). Patient VAS back pain scores did not correlate with the distal level of surgical fusion (*p* = 0.92) and were not worse in patients with longer follow-up (r^2^ = 0.18 and *p* = 0.05). Regression analysis of VAS scores trended to decrease with increased time from surgery (Fig. 1).

**Table 1:** Long-term VAS and Oswestry Disability Scores

Outcome item	Mean value	*P* value distal fusion level	R squared with respect to time	*P* value with respect to time
VAS Back Pain	2.5	0.917	0.177	0.051
Oswestry Disability	15.36	0.715	0.092	0.168

**Fig. 2:**
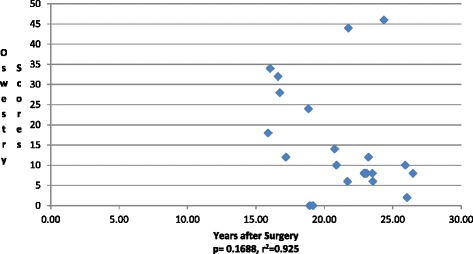
Oswestry Disability Index versus time since surgery

The overall mean ODI of instrumented patients was calculated at 15.36 (Table 1). Six patients reported an ODI over 20. There was no relationship with distal level of fusion and ODI score (*p* = 0.72) (Fig. 2).

**Fig. 3:**
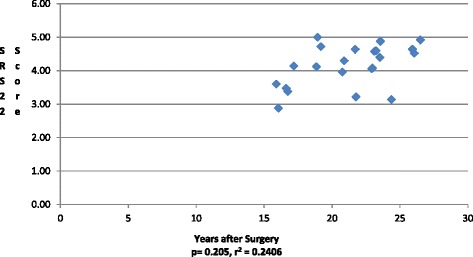
SRS-22 Total Score versus time since surgery

The overall mean SRS-22 of instrumented patients was calculated at 4.15. (Table 2) SRS-22 scores did not correlate with distal level of fusion (*p* = 0.78). None of the SRS-22 subcategories of function (4.47), pain (4.02), self-image (3.75), mental health (4.15), or satisfaction with management (4.34) correlated with distal fusion level. (Table 2) Patient reported SRS-22 total scores improved in patients with longer follow-up (r^2^ = 0.24, *p* = 0.21). (Fig. 3).

**Table 2:** Long-term SRS-22 Scores

Outcome item	Mean value	*P* value distal fusion level	R squared with respect to time	*P* value with respect to time
SRS-22 function	4.47	0.573	0.016	0.574
SRS-22 pain	4.02	0.650	0.098	0.156
SRS-22 self image	3.75	0.681	0.240	0.020
SRS-22 mental health	4.15	0.876	0.184	0.046
SRS-22 satisfaction with management	4.34	0.776	0.295	0.008
SRS-22 Total	4.15	0.779	0.240	0.020

The overall mean SF-36 of instrumented patients was calculated at 72.05. (Table 3) Total SF-36 did not correlate with distal level of fusion (*p* = 0.67). None of the SF-36 subcategories (mean); role limitations due to physical health (87.78), limitations due to emotional well-being (92.58), energy (63.92), emotional well-being (80.68), social function (88.07) and pain (77.84)) correlated with distal fusion level (Table 3). Patient reported SF-36 total scores did not correlate with longer follow-up (r^2^ = 0.67, *p* = 0.03).

**Table 3:** Long-term SF-36 Scores Listed by Domain

Outcome item	Mean value	*P* value distal fusion level	R squared with respect to time	*P* value with respect to time
SF-36- physical function	86.36	0.431	0.021	0.518
SF-36- role limitations due to physical health	87.78	0.555	0.023	0.492
SF-36- limitations due to emotional wellbeing	92.58	0.628	0.151	0.073
SF-36 -energy	63.92	0.973	0.197	0.037
SF-36 -emotional wellbeing	80.68	0.659	0.152	0.072
SF-36 -social function	88.07	0.612	0.153	0.071
SF-36 -pain	77.84	0.711	0.048	0.076
SF-36- general Health	72.05	0.670	0.030	0.435

Table 4 represents the individual fusion levels as well as the outcomes for VAS Back Pain, ODI and SRS-22 Total.

**Table 4:** Level of instrumentation and corresponding functional scores

Patient	Level	VAS-Back pain	SRS-22 Total	ODI
1	T4-T11	3	4.14	12
2	T4-T11	1	4.4	8
3	T4-L1	0	5	0
4	T4-L1	7	3.14	46
5	T2-L2	1	4.6	12
6	T2-L2	8	3.22	44
7	T3-L2	0	4.88	6
8	T3-L2	2	3.6	18
9	T4-L2	0	4.72	0
10	T4-L2	0	4.52	2
11	T4-L2	0	4.64	10
12	T4-L2	8	3.48	32
13	T5-L2	2	4.3	10
14	T5-L2	5	4.12	24
15	T4-L3	0	4.08	8
16	T4-L3	1	4.58	8
17	T4-L3	0	4.92	8
18	T5-L3	5	3.38	28
19	T4-L3	5	2.88	34
20	T5-L4	2	3.96	14
21	T5-L4	4	4.06	8
22	T6-L4	0	4.64	6

## Discussion

The majority of patients who underwent multi-segment spinal fixation for AIS, reported in their telephone interview that they did not have back pain on a daily basis. Of the 22 patients who completed the full assessment of questionnaires sent (ODI, SF-36, VAS, and SRS-22 forms), 15 had a VAS score of less than 3 cm, indicating the overall mean VAS score of the patients to be relatively low. However, six of 22 patients studied reported significant back pain (5 or greater). One third reported moderate back pain VAS (3 or greater). While not statistically significant (*p* = 0.051), patients’ reported VAS back pain trended toward better (lower) scores with increased follow-up (R-squared = 0.17).

The lowest instrumented segment did not appear to be associated with increased back pain at 15–25 year follow-up. VAS, ODI, SRS-22, and SF-36 (including each individual subcategory of SRS-22 and SF-36) did not correlate with lowest instrumented segment. These results are in direct contrast to the studies of Cochran which showed a significant increase in back pain with fusion at or lower than L4 [8]. This discrepancy may be due to the chronology and technique of instrumentation used in the studies, as the patients in our study were treated after 1986 with modern CD instrumentation, where the multilevel fixation and correction technique allow preservation of the sagittal plane. Cochran [8], Bartie [10], and others looked at long-term outcomes of fusions with Harington rod instrumentation. Harrington rods use distraction as the major force for correction of the coronal deformity. However, this mechanism is limited in the correction of the sagittal and apical deformity. At times, Harrington rods can be detrimental in the preservation of the sagittal plane [12]. Historically, this was most problematic in the lower lumbar spine, causing flatback syndrome and lack of correction of the thoracic hump [12]. Long-term results also showed implant breakage of up to 40 % of patients treated with Harrington rods for AIS [12].

Due to the substantial differences in the instrumentation, we believe that studies such as those by Cochran may over-estimate long-term morbidity when applied to populations treated with CD instrumentation principles. From the findings in our study, we suggest less of an emphasis be placed on avoidance of fusions to L4 and L5 levels in patients being surgically treated for AIS with CD instrumentation.

Functional outcome scores (SF-36, SRS-22, and ODI) had varying results in patients with longer follow-up. With some parameters in SRS-22 subcategories of self-image, mental health, satisfaction with management and total SRS score, patients had significantly better scores further out from surgery. SF-36 scores, though not statistically significant, did show improved trends in limitations due to emotional well-being, social function, and pain, as well as, having a significant improvement in the SF-36 subcategory of energy over time. The vast majority of these subcategories involved are psychosocial in nature. We had hypothesized that scores would not worsen over time, but did not anticipate a trend toward improved psychosocial function and less pain (with a trend toward lower VAS scores) over time from surgery.

An obvious limitation of the study is the relatively low response rate. Only half of the patients contacted agreed to complete full functional outcomes. Patients with worse outcomes may have been less inclined to participate, but it is impossible to know exactly how this response rate affected out study outcome. Every attempt to contact study participants was made within the regulations set forth by our IRB. Our study was further limited by the lack of x-rays or clinical follow-up. Clinical outcomes have been associated with factors, such as, pseudoarthrosis and spinal alignment [13]. This is an aspect of long-term follow-up we hope to investigate as we expand this study.

As a final note the authors of this study do receive funding from spinal implant companies in relation to research un-related to the current study. Disclosing potential bias is important. The current study was self-funded which would lend toward the removal of potential bais. These patients had their spinal instrumentation placed 15–25 years prior and were not part of any device investigation funded or unfunded current or present which also adds to the transparency of the study.

## Conclusion

Our study substantiates that low back pain does not correlate with distal level of fusion in patients instrumented with the Cotrel-Dubousset technique for adolescent idiopathic scoliosis. This is in direct contradiction with earlier studies which utilized Harrington instrumentation. We believe this may be due to the newer methods of instrumentation employed and how they affect both planes of the deformity [14]. Our data also shows that functional and general health scores do not correlate with the level of fusion. We also note that back pain and certain functional score subcategories showed a trend for improved results over time from surgery.
